# Microbiological Profile and Antimicrobial Resistance Patterns Among Orthopedic Patients at a Tertiary Care Centre in Uttarakhand, India

**DOI:** 10.7759/cureus.117496

**Published:** 2026-10-05

**Authors:** Diwakar R.D., Rajendra Sharma, Sunita Pandita, Daya Krishna, Kalpana P Date

**Affiliations:** 1 Pharmacology, Veer Chandra Singh Garhwali Government Institute of Medical Science and Research, Uttarakhand, IND; 2 Orthopedics, Veer Chandra Singh Garhwali Government Institute of Medical Science and Research, Uttarakhand, IND; 3 Medical Microbiology, N. K. P. Salve Institute of Medical Sciences & Research Centre and Lata Mangeshkar Hospital, Nagpur, IND

**Keywords:** antibiotic stewardship, antimicrobial resistance, orthopedic anesthesia, orthopedic infections, patients satisfaction

## Abstract

Background

Antimicrobial resistance has complicated the management of bacterial infections among patients admitted to orthopedic wards by limiting effective treatment options and increasing the risk of treatment failure and prolonged hospitalization. This study aimed to describe the distribution of bacterial isolates and antimicrobial susceptibility patterns among patients admitted to an orthopedic ward and to identify clinical factors associated with multidrug-resistant (MDR) isolates.

Methods

This hospital-based cross-sectional study included 96 patients admitted to the orthopedic ward who had culture-positive clinical specimens and met the study eligibility criteria. Demographic and clinical characteristics, specimen types, bacterial isolates, previous antibiotic exposure, hospitalization history, implant status, and antimicrobial susceptibility profiles were recorded. MDR was defined as non-susceptibility to at least one agent in three or more antimicrobial categories.

Results

The mean age of the patients was 49.4 ± 18.8 years, and 58 (60.4%) were male. Urine was the most common specimen, comprising 50 (52.1%) samples. *Escherichia coli* was the predominant isolate, identified in 28 (29.2%) patients, followed by *Staphylococcus aureus* in 20 (20.8%). High rates of resistance were observed to cefazolin, cefepime, ciprofloxacin, and trimethoprim-sulfamethoxazole. MDR isolates were identified in 42 (43.8%) patients. MDR isolation was significantly associated with age >60 years, diabetes mellitus, orthopedic implant status, previous antibiotic exposure, previous hospitalization, and Gram-negative isolates. After adjustment for potential confounders, previous antibiotic exposure (adjusted OR=3.12, 95% CI: 1.20-8.16; p=0.020) and previous hospitalization (adjusted OR=2.73, 95% CI: 1.00-7.41; p=0.049) remained independently associated with MDR isolation. Patients with MDR isolates had a longer mean hospital stay than those without MDR isolates (14.1 ± 5.8 versus 8.9 ± 4.5 days; p<0.001); however, the cross-sectional design precludes determination of the temporal direction of this association.

Conclusions

Antimicrobial resistance and MDR isolates were common among patients admitted to the orthopedic ward. Previous antibiotic exposure and hospitalization were independently associated with MDR isolation, highlighting the importance of antimicrobial stewardship and appropriate microbiological evaluation in this population.

## Introduction

Antimicrobial resistance has become one of the most important global public health challenges currently facing the health sector, diminishing the efficacy of widely used antibiotics and making the treatment of bacterial infections more challenging [1]. Drug-resistant infections are linked to increased disability and death, longer hospital stays, increased health care costs, and longer illnesses. In 2021, bacterial antimicrobial resistance contributed to over 4.7 million deaths globally, and in 2023, around 16% of all bacterial infections identified in laboratories were resistant to antibiotic treatment [2]. Across many pathogen/antibiotic pairs, resistance has been on the rise, especially in the Eastern Mediterranean and South-East Asian regions [3]. Infections in the bone, joints, surgical wounds, fractures, and implants are commonly hard to cure, making antimicrobial resistance particularly problematic in orthopedic surgery. Orthopedic patients often need surgical fixation, joint replacement, repeated debridement, antimicrobial therapy, and/or a prolonged period of hospital stay [4]. Microbial adherence and biofilm formation are also facilitated by the presence of plates and screws, the prosthesis itself, and any other implants. Biofilms are associated with sub-optimal response to antimicrobial agents and host immune responses, leading to persistent or recurrent infection despite apparent appropriate antimicrobial treatment [5].

There are different organisms that cause orthopedic infections depending on the type of procedure, location, healthcare environment, and prior exposure to antibiotics. *Staphylococcus aureus* and coagulase-negative staphylococci continue to be serious pathogens of surgical-site, fracture-related, and prosthetic-joint infections [6]. In recent years, however, Gram-negative organisms like *Escherichia coli*, *Klebsiella pneumoniae*, *Pseudomonas aeruginosa*, and *Acinetobacter* species have been identified, especially in trauma patients, open fractures, and in resource-limited environments with prolonged hospital stays. There have also been studies demonstrating significant differences in the microbiological flora of fracture-related infections and periprosthetic joint disease (PJD), suggesting that one empirical antibiotic treatment should not be used in all orthopedic patients [7]. Furthermore, the prevalence of methicillin-resistant *S. aureus* (MRSA), multidrug-resistant Gram-negative bacilli (MDR-GNB), extended-spectrum beta-lactamase (ESBL) producing organisms, and carbapenem-resistant bacteria (CRB) has added to the challenges of orthopedic infection management [8]. A low-resource group identified the Gram-negative bacteria as major causative organisms of fracture infection, and their susceptibility to several commonly employed antimicrobials was limited. The empirical selection of antibiotics depends on the susceptibility characteristics of the organisms, with isolates from different geographical or healthcare areas exhibiting varying patterns of susceptibility [9]. Only a few isolates of Gram-negative organisms were susceptible to treatment rates of more than 50%, suggesting an increased risk of selecting an empirical antibiotic regimen based on isolates from different regions or in different healthcare environments. Similarly, an increase in treatment failure, multiple surgical procedures, longer antimicrobial treatment duration, and reduced functional outcomes have been associated with resistant PJD [10].

Resistance is a complex process that is seen among orthopedic patients due to several factors such as improper initial antibiotic use, unnecessary prophylactic antibiotics, length of treatment, prior exposure to antibiotics, repeat admissions, inadequate infection control practices, and delayed microbiological diagnosis [11]. A surveillance study in India of orthopedic infections concluded that surgical-site infection rates varied over time and highlighted the need to keep track of bacterial isolates, antibiotic susceptibility, and antibiotic prescribing patterns. Therefore, local surveillance is necessary, as resistance patterns may vary significantly between hospitals and may be variable within a hospital over time. Antimicrobials represent one of the top prescription drug classes used today in clinical practice [12]. This broad and often empirical antibiotic use has, however, resulted in the worrying trend of antimicrobial resistance becoming a worldwide public health issue. Treatment is started empirically in hospitals before the microbiological culture and sensitivity report, particularly in developing countries such as India. These were justified in clinical situations but have led to the selection and dissemination of resistant strains of bacteria [13].

This study aimed to describe the distribution of bacterial isolates and antimicrobial susceptibility patterns among patients admitted to an orthopedic ward and to identify clinical factors associated with MDR isolates.

## Materials and methods

Study design and setting

This observational, cross-sectional study was conducted in the Department of Pharmacology in collaboration with the Department of Orthopedics at Veer Chandra Singh Garhwali Government Institute of Medical Science and Research (VCSGGIMS&R), Srinagar, Pauri Garhwal, Uttarakhand, from December 2024 to May 2025. The study was approved by the Institutional Ethics Committee of VCSGGIMS&R (IEC No. MC/IEC/2024/61).

Selection criteria and sample size

During the predefined study period, all eligible patients admitted to the orthopedic ward who underwent clinical sampling, had bacterial growth on culture, and received antimicrobial therapy were recruited consecutively. The study population therefore comprised culture-positive patients admitted to the orthopedic ward and was not restricted to patients with confirmed orthopedic-site infections. Because the study was designed as a hospital-based observational study covering a fixed six-month period, no formal a priori sample size or power calculation was performed. A total of 96 eligible patients were included in the final analysis.

Patients admitted to the orthopedic ward who underwent clinical sampling and yielded bacterial growth on culture were eligible for inclusion. The clinical indications for sampling included suspected surgical-site, fracture-related, bone/joint, implant-associated, or other infections identified during the hospital stay and infected open fractures. Because urine and other non-orthopedic specimens were also included, a positive culture was not considered synonymous with an orthopedic-site infection.

A total of 12 patients with contaminated specimens, 16 with incomplete clinical and microbiological data, 14 with fungal or mycobacterial infections, and one with culture-negative infections were excluded. Only the first organism encountered in an infectious episode was added if multiple cultures from the same patient were positive for the same organism.

Data sources and variables

Data for demographic and clinical characteristics were collected on a structured data collection form. Collected variables were age, gender, comorbidities, anatomic site and type of infection, history of trauma or orthopedic surgery, presence of an implant, prior hospitalization, prior antibiotic exposure, specimen type, isolated organism, and antimicrobial susceptibility profile. Previous antibiotic exposure was defined a priori as receipt of systemic antimicrobial therapy within the 90 days preceding collection of the clinical specimen. Exposure was recorded as a binary variable (yes/no). Detailed information regarding antimicrobial class, number of courses, duration, dose, and indication was not consistently available in the study records and therefore was not included in the primary analysis.

All clinical specimens such as deep wound swabs, pus, tissue, bone, synovial fluid, and aspirates were collected aseptically. Samples were collected before the start or change of antibiotic treatment whenever possible. When a suitable deep tissue or fluid specimen was available, superficial swabs were not taken. Urine specimens were analyzed as a separate specimen category. Urine specimens were included because the study population was defined by admission to the orthopedic ward rather than by infection site. Urinary isolates were analyzed as culture-positive isolates and interpreted separately from confirmed orthopedic-site infections.

Samples were promptly sent to the microbiology laboratory for analysis following standard microbiology lab procedures. Appropriate culture media were used, and specimens were cultured aerobically or anaerobically depending on the type of specimen and clinical indication. Bacterial isolates were characterized by colony morphology, Gram staining, biochemical characteristics, and automated identification systems, if available.

Antimicrobial susceptibility testing

Antimicrobial susceptibility testing was performed according to the Kirby-Bauer disc diffusion method on Mueller-Hinton agar. Where clinically indicated and laboratory facilities permitted, minimum inhibitory concentration testing was also performed. Susceptibility results were interpreted according to the Clinical and Laboratory Standards Institute (CLSI) guidelines in use during the study period. The antimicrobial agents tested varied according to the bacterial species and clinical indication and included agents from the penicillin, cephalosporin, carbapenem, aminoglycoside, fluoroquinolone, macrolide, glycopeptide, tetracycline, oxazolidinone, and polymyxin classes. An antimicrobial agent was considered ‘not tested’ when susceptibility testing was not performed for a particular organism; such agents were not included in the denominator for resistance-rate calculations or comparative statistical analyses. Detailed laboratory records specifying the exact CLSI edition, antimicrobial disc concentrations, and quality-control strains were not available for retrospective verification.

Isolates were categorized as susceptible, intermediate, or resistant according to the applicable CLSI interpretive criteria. Intermediate and resistant categories were combined a priori and reported as non-susceptible for the purposes of statistical analysis to give conservative estimates of non-susceptibility. MRSA was detected by testing for cefoxitin susceptibility. The production of ESBLs and carbapenem resistance was determined with the standard phenotypic methods for Gram-negative bacteria. Multidrug resistance was considered as non-susceptibility to at least one agent in three or more antimicrobial categories. The primary outcomes were the distribution of bacterial isolates and their antimicrobial susceptibility patterns. MDR status was analyzed as a secondary outcome in relation to clinical and microbiological characteristics. Secondary variables analyzed were the presence of MDR organisms and the difference in resistance patterns based on type of infection, Gram stain, implant status, prior antibiotic use, and other clinical variables.

Statistical analysis

IBM SPSS Statistics for Windows, Version 26 (Released 2018; IBM Corp., Armonk, NY, USA) was used to analyze the data. Continuous variables were tested for normality and are presented as mean ± SD or median (interquartile range) as appropriate. Categorical variables were described in terms of number and percentage. The proportion of resistant isolates to the total number of isolates/subtyped against a given antibiotic was computed as antimicrobial resistance rates. The chi-square test or Fisher's exact test was used to assess associations between antimicrobial resistance and clinical characteristics, as appropriate. An independent-samples t-test or Mann-Whitney U test was used for continuous variables according to their distribution. Only those variables that were clinically relevant and/or statistically significant in the univariable analyses were included in the multivariable logistic regression to determine independent factors associated with MDR infection. Odds ratios (ORs) were reported with 95% confidence intervals (CIs). A two-sided p-value < 0.05 was considered significant.

## Results

Demographic and clinical characteristics of patients admitted to the orthopedic ward are depicted in Table 1. The mean age was 49.4 ± 18.8 years, with the largest proportion of patients aged >60 years, comprising 30 (31.2%) patients, followed by those aged 20-30 years with 27 (28.1%), 31-40 years with 21 (21.9%), and 41-60 years with 18 (18.8%). Of the 96 patients, 58 (60.4%) were male, and 38 (39.6%) were female. Diabetes mellitus was present in 30 (31.2%) patients, while 28 (29.2%) had an orthopedic implant. Prior antibiotic use within 90 days was reported by 44 (45.8%) patients, and 34 (35.4%) had a history of previous hospitalization. The mean duration of hospital stay was 11.2 ± 5.7 days.

**Table 1:** Demographic and clinical characteristics of patients admitted to the orthopedic ward (n=96) Values are presented as mean ± SD or n (%). SD: standard deviation

Variable	Value
Age, years, mean ± SD	49.4 ± 18.8
Age group, n (%)	
20-30 years	27 (28.1)
31-40 years	21 (21.9)
41-60 years	18 (18.8)
>60 years	30 (31.2)
Sex, n (%)	
Male	58 (60.4)
Female	38 (39.6)
Diabetes mellitus, n (%)	
Yes	30 (31.2)
No	66 (68.8)
Orthopedic implant, n (%)	
Yes	28 (29.2)
No	68 (70.8)
Antibiotic exposure during the previous 90 days, n (%)	
Yes	44 (45.8)
No	52 (54.2)
Previous hospitalization, n (%)	
Yes	34 (35.4)
No	62 (64.6)
Duration of hospitalization days, mean ± SD	11.2 ± 5.7

The microbiological profile of bacterial isolates among culture-positive patients admitted to the orthopedic ward is shown in Table 2. Urine specimens were the most common clinical samples, accounting for 50 (52.1%), followed by wound or soft tissue specimens, 20 (20.8%); blood cultures, 10 (10.4%); device-associated specimens, 8 (8.3%); and other specimens, including synovial fluid, 8 (8.3%). *E. coli* was the most frequently isolated organism, identified in 28 (29.2%) patients, followed by *S. aureus* in 20 (20.8%), *Klebsiella* species in 10 (10.4%), *Pseudomonas* species in 8 (8.3%), *Enterococcus* species in 6 (6.2%), and coagulase-negative staphylococci in 5 (5.2%) patients.

**Table 2:** Microbiological profile of bacterial isolates among culture-positive patients admitted to the orthopedic ward Values are presented as n (%).

Variable	n (%)
Clinical specimen	
Urine	50 (52.1)
Wound/soft tissue	20 (20.8)
Blood	10 (10.4)
Device-associated specimen	8 (8.3)
Other specimens, including synovial fluid	8 (8.3)
Bacterial isolate	
Escherichia coli	28 (29.2)
Staphylococcus aureus	20 (20.8)
*Klebsiella* species	10 (10.4)
*Pseudomonas* species	8 (8.3)
*Enterococcus* species	6 (6.2)
Coagulase-negative staphylococci	5 (5.2)
Other bacterial isolates	19 (19.8)
Organism category	
Gram-negative organisms	54 (56.2)
Gram-positive organisms	42 (43.8)

Because urine specimens constituted more than half of all samples, the overall microbiological profile should be interpreted as representing culture-positive patients admitted to the orthopedic ward rather than exclusively orthopedic-site infections.

Table 3 shows that high resistance rates to first- and fourth-generation cephalosporins were observed across the major pathogens. Cefazolin resistance ranged from 60.0% (6/10) in *Klebsiella* species to 85.0% (17/20) in *S. aureus*, while cefepime resistance ranged from 70.0% (7/10) to 80.0% (16/20), respectively. Ciprofloxacin resistance was highest in *E. coli* at 64.3% (18/28) and lowest in *S. aureus* at 40.0% (8/20).

**Table 3:** Antimicrobial resistance patterns of the principal bacterial isolates Values are presented as resistant, n (%). The denominator for each percentage corresponds to the number of isolates of the respective bacterial species tested for that antimicrobial agent. ‘Not tested’ indicates that susceptibility testing was not performed for that organism-antimicrobial combination and was excluded from the corresponding denominator and statistical analysis. *E. coli*: *Escherichia coli*; *S. aureus*: *Staphylococcus aureus*

Antimicrobial agent	*E. coli* (n=28), resistant n (%)	*S. aureus* (n=20), resistant n (%)	*Klebsiella* spp. (n=10), resistant n (%)	Statistical test	p-value
Cefazolin	19/28 (67.9)	17/20 (85.0)	6/10 (60.0)	χ²=2.65	0.266
Cefepime	21/28 (75.0)	16/20 (80.0)	7/10 (70.0)	χ²=0.39	0.824
Ciprofloxacin	18/28 (64.3)	8/20 (40.0)	5/10 (50.0)	χ²=2.82	0.244
Trimethoprim-sulfamethoxazole	17/28 (60.7)	13/20 (65.0)	6/10 (60.0)	χ²=0.11	0.945
Carbapenems	3/28 (10.7)	Not tested	2/10 (20.0)	Fisher’s exact test	0.592
Nitrofurantoin	4 (14.3)	Not tested	Not tested	Not applicable	Not applicable
Linezolid	Not tested	1 (5.0)	Not tested	Not applicable	Not applicable
Clindamycin	Not tested	2 (10.0)	Not tested	Not applicable	Not applicable

As per Table 4, overall, 42 (43.8%) patients had MDR isolates. The highest proportion of MDR isolates was observed among patients with *Klebsiella* species (70.0%), followed by *Pseudomonas* species (62.5%), *E. coli* (57.1%), and *S. aureus* (45.0%), with a significant association between bacterial species and MDR status (p=0.014). Patients aged >60 years had a significantly higher likelihood of MDR isolation compared with younger patients (60.0% vs. 36.4%; OR=2.63, 95% CI: 1.08-6.37; p=0.030).

**Table 4:** Distribution and factors associated with multidrug-resistant isolates among patients admitted to the orthopedic ward Values are presented as n (%) unless otherwise indicated. Reference categories are indicated in the table. OR: odds ratio; CI: confidence interval; MDR: multidrug resistance

Variable	MDR, n (%)	Non-MDR, n (%)	Effect estimate	Test statistic	p-value
Orthopedic implant					
Present	18 (64.3)	10 (35.7)	OR: 3.30 (95% CI: 1.32-8.27)	χ² = 6.77	0.009
Absent	24 (35.3)	44 (64.7)	Reference		
Previous antibiotic exposure					
Yes	27 (61.4)	17 (38.6)	OR: 3.92 (95% CI: 1.67-9.20)	χ² = 10.24	0.001
No	15 (28.8)	37 (71.2)	Reference		
Previous hospitalization					
Yes	21 (61.8)	13 (38.2)	OR: 3.15 (95% CI: 1.32-7.52)	χ² = 6.94	0.008
No	21 (33.9)	41 (66.1)	Reference		
Organism category					
Gram-negative	30 (55.6)	24 (44.4)	OR: 3.13 (95% CI: 1.32-7.37)	χ² = 6.99	0.008
Gram-positive	12 (28.6)	30 (71.4)	Reference		
Specimen category					
Urine	25 (50.0)	25 (50.0)	OR: 1.71 (95% CI: 0.75-3.86)	χ² = 1.66	0.198
Non-urine	17 (37.0)	29 (63.0)	Reference		
Continuous variables					
Age, years, mean ± SD	55.6 ± 17.4	44.6 ± 18.6	Mean difference: 11.0 years	t = 2.96	0.004
Hospital stay, days, mean ± SD	14.1 ± 5.8	8.9 ± 4.5	Mean difference: 5.2 days	t = 4.95	<0.001
Overall MDR status	42 (43.8)	54 (56.2)	-	-	-

After adjustment for potential confounders, previous antibiotic exposure remained an independent factor associated with MDR isolation, increasing the odds more than threefold (adjusted OR=3.12, 95% CI: 1.20-8.16; p=0.020). Previous hospitalization was also independently associated with MDR isolation (adjusted OR=2.73, 95% CI: 1.00-7.41; p=0.049), as evident in Table 5.

**Table 5:** Multivariable logistic regression analysis of predictors of multidrug-resistant infection Dependent variable: multidrug-resistant infection. Nagelkerke R² = 0.365; -2 log likelihood = 103.48; Hosmer-Lemeshow χ² = 10.57, p = 0.158; overall classification accuracy = 72.9%; area under the receiver operating characteristic curve = 0.802.

Predictor	β coefficient	Standard error	Wald statistic	Adjusted OR (95% CI)	p-value
Age >60 years	0.695	0.530	1.72	2.00 (0.71-5.66)	0.190
Diabetes mellitus	0.932	0.540	2.98	2.54 (0.88-7.31)	0.084
Orthopedic implant present	0.965	0.533	3.28	2.62 (0.92-7.47)	0.071
Previous antibiotic exposure	1.139	0.489	5.42	3.12 (1.20-8.16)	0.020
Previous hospitalization	1.003	0.510	3.87	2.73 (1.00-7.41)	0.049
Gram-negative isolate	0.927	0.503	3.40	2.53 (0.94-6.77)	0.066
Constant	-2.081	0.611	11.61	0.12 (0.04-0.41)	0.001

## Discussion

The present cross-sectional observational study evaluated the pattern of antimicrobial resistance among patients admitted to the orthopedic ward at a tertiary care hospital in Pauri Garhwal, Uttarakhand. Gram-negative organisms slightly outnumbered Gram-positive organisms, with *E. coli* and *S. aureus* being the predominant pathogens. High resistance rates were observed to cefazolin, cefepime, ciprofloxacin, and trimethoprim-sulfamethoxazole, whereas resistance to carbapenems, linezolid, and clindamycin was comparatively lower. Previous antibiotic exposure and hospitalization were independently associated with MDR isolation, and patients with MDR isolates had significantly longer hospital stays. These findings highlight the presence of antimicrobial-resistant organisms across different clinical specimen types in the orthopedic patient population.

Urine specimens constituted the largest proportion of clinical samples (52.1%), which substantially influences interpretation and may partly explain the predominance of *E. coli* in the overall bacterial profile. A previous study of UTIs among orthopedic patients similarly identified *E. coli* and *K. pneumoniae* as the predominant Gram-negative pathogens, with substantial resistance to extended-spectrum cephalosporins and quinolones [13]. However, the findings should be interpreted cautiously because a positive urine culture alone does not establish symptomatic urinary tract infection (UTI). Asymptomatic bacteriuria is common among older adults, catheterized patients, and hospitalized surgical patients, and unnecessary urine testing may contribute to inappropriate antibiotic use. In this context, a previous study found that discontinuing routine urinalysis before elective orthopedic surgery did not increase catheter-associated UTI or surgical-site infection rates and was associated with reduced antibiotic use [14].

Although *E. coli* was the most frequently isolated organism overall, *S. aureus* was the predominant Gram-positive pathogen, particularly in wound and device-associated infections. This finding is consistent with previous orthopedic literature. A multicountry study of 2,038 postoperative orthopedic infections identified *S. aureus* as the most common Gram-positive organism, while *Klebsiella *species was the predominant Gram-negative organism [15]. Differences between these findings and the present study may reflect the inclusion of urine and blood cultures in the current analysis, whereas many orthopedic studies focus primarily on deep wound, bone, or implant-associated specimens. The predominance of Gram-negative organisms is clinically relevant because empirical antimicrobial treatment of orthopedic infections has traditionally focused largely on staphylococcal pathogens [16]. Recent studies of fracture-related infections have also demonstrated that Gram-negative bacilli contribute substantially to both monomicrobial and polymicrobial infections, with considerable variation between institutions and geographical regions [17].

High resistance to commonly used cephalosporins was observed in the present study. Cefepime resistance among the three major organisms ranged from 70% to 80%, while cefazolin resistance ranged from 60% to 85%. Resistance to ciprofloxacin and trimethoprim-sulfamethoxazole was also substantial, particularly among *E. coli*. Similar resistance patterns have been reported among Gram-negative organisms isolated from orthopedic and urinary infections in hospitalized orthopedic patients, raising concerns regarding empirical use of these agents without microbiological confirmation [18]. Although carbapenem resistance was comparatively lower among *E. coli* and *Klebsiella* species, their use should be guided by culture and susceptibility results and reserved for appropriate clinical indications. Importantly, cefazolin resistance should not be used as a surrogate for methicillin resistance in *S. aureus*; methicillin resistance should be determined using cefoxitin or an appropriate MIC-based method [19].

The proportion of patients with MDR isolates was 43.8%, indicating a substantial antimicrobial resistance burden among culture-positive patients admitted to the orthopedic ward. Previous antibiotic exposure and hospitalization remained independently associated with MDR isolation after adjustment for potential confounders. These findings emphasize the importance of antimicrobial stewardship, appropriate microbiological investigation, and careful selection of empirical therapy in orthopedic patients, particularly those with prior antibiotic exposure or hospitalization.

Limitations

This study has several limitations. It was conducted at a single tertiary care centre with a relatively small sample size, which may limit the generalizability of the findings. The inclusion of different infection sites and specimen types, particularly urine specimens, may have influenced the overall distribution of bacterial isolates and resistance patterns. 

Prior antibiotic exposure was assessed as a binary variable based on systemic antimicrobial receipt within the preceding 90 days. Detailed information regarding antimicrobial class, number of courses, duration, dose, and indication was not consistently available, which may have resulted in exposure misclassification and limited assessment of dose-response or class-specific associations.

The clinical definition of infection was based on the clinical and microbiological information available in the study records. Culture-negative cases and standardized differentiation between colonization, asymptomatic bacteriuria, symptomatic infection, and confirmed orthopedic-site infection were not available for all patients. Consequently, the findings should be interpreted as describing culture-positive patients admitted to the orthopedic ward rather than a population exclusively comprising confirmed orthopedic-site infections.

Most antimicrobial susceptibility testing was based on phenotypic methods, with limited availability of MIC-based or molecular characterization of resistance mechanisms. Information regarding antibiotic dose and duration, adherence, surgical procedures, and long-term clinical outcomes was also limited.

The number of MDR events was limited, which may have reduced the precision and stability of the multivariable logistic regression estimates. The regression model was therefore considered exploratory, and the observed associations should not be interpreted as validated prediction estimates. External validation in larger and independent cohorts is required.

Finally, the observational design precludes establishing causal relationships between the identified factors and MDR isolation. Larger multicentre prospective studies incorporating standardized specimen collection, comprehensive susceptibility testing, molecular characterization, and longitudinal follow-up are warranted to validate these findings.

## Conclusions

In conclusion, this single-centre cross-sectional study of culture-positive patients admitted to an orthopedic ward demonstrated substantial antimicrobial resistance, with *E. coli *and *S. aureus* being the most frequently identified organisms. Marked resistance was observed to commonly used cephalosporins, ciprofloxacin, and trimethoprim-sulfamethoxazole. Previous antibiotic exposure and previous hospitalization were independently associated with MDR isolation after adjustment for selected clinical factors. However, the high proportion of urine specimens and the absence of standardized clinical criteria for distinguishing symptomatic urinary infection from asymptomatic bacteriuria or colonization limit interpretation of the overall microbiological profile as representative of orthopedic-site infections. These findings support local microbiological surveillance and culture-guided antimicrobial stewardship among patients receiving orthopedic care.
